# Survey of poliovirus antibodies in Borno and Yobe States, North-Eastern Nigeria

**DOI:** 10.1371/journal.pone.0185284

**Published:** 2017-09-26

**Authors:** Mustapha Modu Gofama, Harish Verma, Hamisu Abdullahi, Natalie A. Molodecky, Kehinde T. Craig, Utibe-Abasi Urua, Mohammed Ashir Garba, Mohammed Arab Alhaji, William C. Weldon, M. Steven Oberste, Fiona Braka, Ado J. G. Muhammad, Roland W. Sutter

**Affiliations:** 1 University of Maiduguri Teaching Hospital, Maiduguri, Nigeria; 2 World Health Organization, Geneva, Switzerland; 3 World Health Organization, Abuja, Nigeria; 4 National Primary Health Care Development Agency, Abuja, Nigeria; 5 Centers for Disease Control and Prevention, Atlanta, Georgia, United States of America; Public Health England, UNITED KINGDOM

## Abstract

**Background:**

Nigeria remains one of only three polio-endemic countries in the world. In 2016, after an absence of 2 years, wild poliovirus serotype 1 was again detected in North-Eastern Nigeria. To better guide programmatic action, we assessed the immunity status of infants and children in Borno and Yobe states, and evaluated the impact of recently introduced inactivated poliovirus vaccine (IPV) on antibody seroprevalence.

**Methods and findings:**

We conducted a facility-based study of seroprevalence to poliovirus serotypes 1, 2 and 3 among health-seeking patients in two sites each of Borno and Yobe States. Enrolment was conducted amongst children 6–9 and 36–47 months of age attending the paediatrics outpatient department of the selected hospitals in the two states between 11 January and 5 February 2016. Detailed demographic and immunization history of the child was taken and an assessment of the child’s health and nutritional state was conducted via physical examination. Blood was collected to test for levels of neutralizing antibody titres against the three poliovirus serotypes. The seroprevalence in the two age groups, potential determinants of seropositivity and the impact of one dose of IPV on humoral immunity were assessed. A total of 583 subjects were enrolled and provided sufficient quantities of serum for testing. Among 6-9-month-old infants, the seroprevalence was 81% (74–87%), 86% (79–91%), and 72% (65–79%) in Borno State, and 75% (67–81%), 74% (66–81%) and 69% (61–76%) in Yobe States, for serotypes-1, 2 and 3, respectively. Among children aged 36–47 months, the seroprevalence was >90% in both states for all three serotypes, with the exception of type 3 seroprevalence in Borno [87% (80–91%)]. Median reciprocal anti-polio neutralizing antibody titers were consistently >900 for serotypes 1 and 2 across age groups and states; with lower estimates for serotype 3, particularly in Borno. IPV received in routine immunization was found to be a significant determinant of seropositivity and anti-polio neutralizing antibodies among 6-9-month-old infants for serotypes 1 and 3, but demonstrated a non-significant positive association for serotype 2. Children receiving IPV through SIAs demonstrated significantly higher anti-polio neutralizing antibodies for serotypes 1 and 3.

**Conclusions:**

The seroprevalence to poliovirus remains suboptimal in both Borno and Yobe States in Nigeria. The low seroprevalence facilitated the continued transmission of both wild serotype 1 and serotype 2 circulating vaccine-derived poliovirus detected in Borno State in 2016. Further efforts are necessary to improve the immunity status of these populations to ensure sufficient population immunity to interrupt transmission.

## 1. Introduction

Currently, three countries remain endemic for poliomyelitis–Pakistan, Afghanistan and Nigeria. In 2016, only 37 cases of serotype-1 wild poliomyelitis (WPV1) were reported globally, the lowest annual number since the Global Polio Eradication Initiative (GPEI) was formed in 1988 [1]. Many additional achievements have been attained including the last reported naturally occurring isolation of serotype 2 wild poliovirus in 1999 and the last reported case of serotype 3 poliomyelitis in 2012. Moreover, since 2014, all serotype 1 poliomyelitis cases have been reported from the three endemic countries, with the last reported non-endemic case in Africa in August 2014 (Somalia).

There have been substantial achievements in Nigeria with a more than 95% reduction in annual cases over the past five years, and no WPV1 cases reported in Nigeria between July 2014 and July 2016. However, after two years with an absence of reported WPV1 cases in Nigeria, four cases were reported from Borno State [2]. These cases were genetically linked to WPV1 circulation from 2012, indicating failures in surveillance in this area for at least four years. In addition, a serotype 2 circulating vaccine-derived poliovirus (cVDPV2) isolate was reported from an environmental surveillance sample in the accessible areas of Borno State collected in March 2016 [3]. This cVDPV2 isolate was the first to be reported in Nigeria since September 2015. Genetic sequencing suggested that this isolate had been in circulation for at least two years and originated from circulation in bordering Chad. Long-standing undetected transmission of WPV1 and cVDPV2 clearly indicates surveillance gaps in this region. Borno is the only Nigerian State to have reported WPV1 cases since 2014.

**Fig 1:**
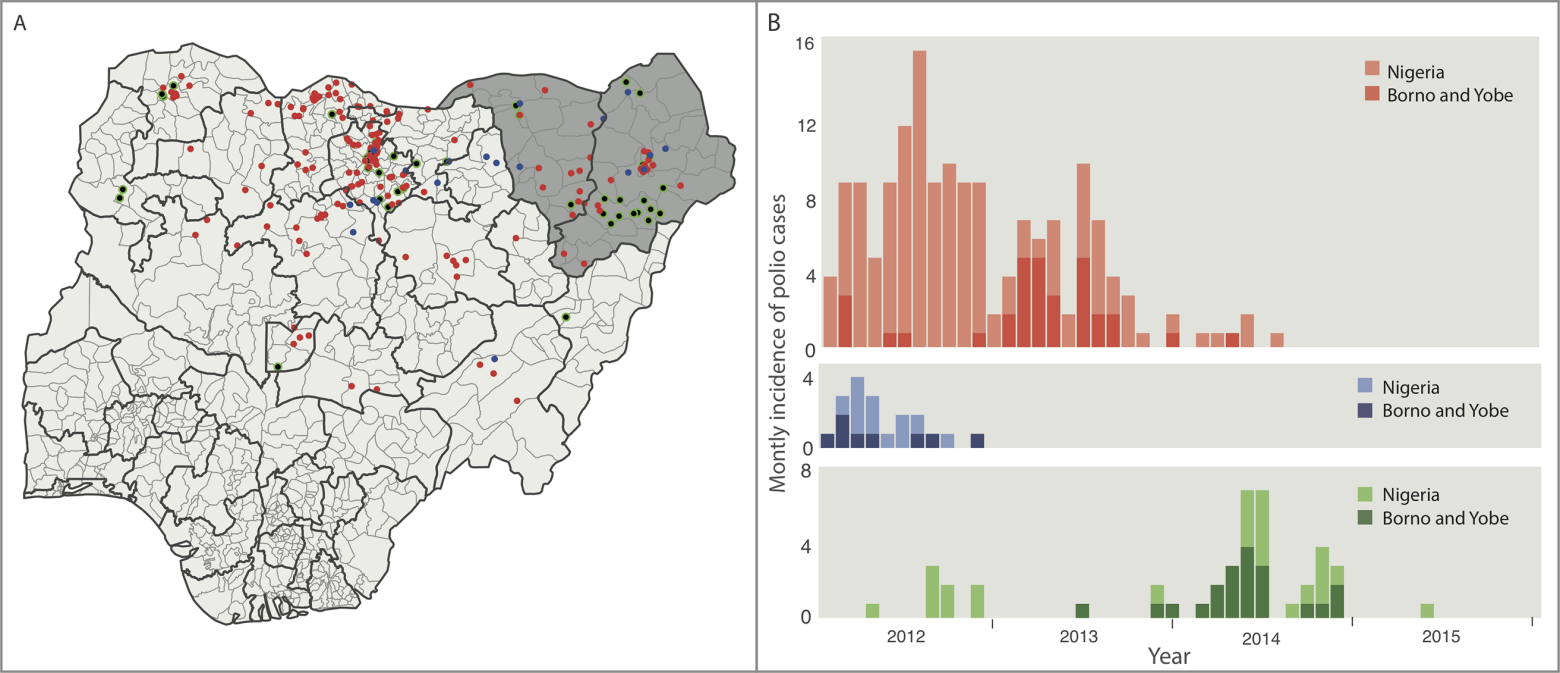
Spatio-temporal distribution of wild poliovirus (WPV) type 1 and 3 and cVDPV2 cases between 2012 and 2015 in Nigeria. Colour of dots and bars correspond to virus type, with red, blue and green corresponding to WPV serotypes 1 and 3 and cVDPV2 cases, respectively. (A) Spatial distribution of WPV serotypes 1 and 3 and cVDPV2. Grey shading in Borno and Yobe States. (B) Monthly incidence of WPV serotypes 1 and 3 and cVDPV2. Darker bars correspond to number of cases from Borno and Yobe.

North-Eastern Nigeria has historically been at high risk of poliovirus transmission and substantially contributed to the national total number of polio cases (Fig 1). This region, including large parts of Borno and some areas in Yobe and Adamawa states, has been affected by Boko Haram insurgency since 2009 [4] which has greatly impacted the quality of field operations for vaccination and surveillance. Accessibility, security issues and large-scale population movements have contributed to the complexity of the field operations and have led to substantial barriers to surveillance and a lack of confidence about the achievements in polio eradication. Despite surveillance challenges, Borno state reported 17 WPV1 cases and 4 cVDPV cases in 2013, and 14 cVDPV2 cases in 2014. Similarly, Yobe detected 8 WPV1 cases in 2013, and 1 WPV1 case and 3 cVDPV2 cases in 2014. Large areas are still inaccessible, with estimates of completely inaccessible populations ranging from 400,000 to 800,000 [4]. Despite some evidence for improvements over the past 6 months, substantial challenges with security, accessibility and surveillance remain.

Challenges apart, like most Northern states in Nigeria, Borno and Yobe have, over the years, implemented multiple supplementary immunization activities (SIAs) with oral poliovirus vaccines (OPV) called Immunization Plus Days (IPDs). In order to reach and access missing populations with OPV, the polio program has adopted many innovations, such as “Hit and Run” vaccination, vaccination at transit points, covering internally displaced population camps, and improving vaccine acceptance in hesitant populations. In addition to vaccination with trivalent OPV (tOPV) or bivalent OPV (bOPV), one dose of inactivated poliovirus vaccine (IPV) was added to the OPV campaign in June-August 2014 to capitalise on the boosting effect of IPV. Subsequently, in February 2015, as a requirement prior to global OPV2 withdrawal in April 2016, one dose IPV at DTP3 contact was added to routine immunization (RI).

Given the utility of seroprevalence data in guiding program interventions, as demonstrated in other endemic states [5–7] and countries [8–10], and the importance of North-Eastern Nigeria in securing a polio-free world, the Nigeria Expert Review Committee (ERC) for polio eradication recommended a polio seroprevalence survey to be conducted in the region. Here, we present the results of a health facility-based seroprevalence survey conducted in children aged 6–9 and 36–47 months of age in Borno and Yobe states.

## 2. Methods

### 2.1. Study design

A health facility cross-sectional design was used in the surveys for Borno and Yobe states. Enrolment was conducted amongst children attending the paediatrics outpatient department (OPD) of the selected hospitals in the two states. Seroprevalence levels were assessed in two age groups: 6–9 and 36–47 months of age.

### 2.2. Objectives

The primary study objectives were to: i) estimate seroprevalence to poliomyelitis (based on anti-polio neutralizing antibodies against serotypes 1, 2 and 3) in children 6–9 and 36–47 months of age, from Borno and Yobe states, Nigeria; ii) evaluate associated factors for seroprevalence levels; and iii) assess IPV coverage and compare seroprevalence in IPV with non-IPV groups. Secondary objectives (inspired by peer-review) were to: i) assess reciprocal anti-polio neutralizing antibodies against serotypes 1, 2 and 3 in children 6–9 and 36–47 months of age, from Borno and Yobe states; and ii) compare the anti-polio neutralizing antibodies in the IPV with non-IPV groups.

### 2.3. Selection of study area

Borno and Yobe states are located in the North-Eastern part of Nigeria–an area endemic for WPV1. The region has also faced insurgency for the past 7 years which has resulted in both local and international migration of people with major consequences for polio risk. The immunity profile in these states may have implications not just for Nigeria but for the neighbouring countries in the Lake Chad sub-region.

### 2.4. Selection of study sites

The following health facilities were selected for the study: 1) University of Maiduguri Teaching Hospital, Borno; 2) State Specialist Hospital Maiduguri, Borno; 3) Federal Medical Centre Nguru, Yobe; and 4) General Sani Abacha Specialist Hospital Damaturu, Yobe. The sites were selected to ensure representation from different populations of interest; Borno sites represented Maiduguri and Jere urban areas where many internally displaced persons (IDPs) live in camps and within host communities. The catchment areas of Yobe hospitals included Damaturu which had a substantial exodus due to insecurity and violence and Nguru with a relatively stable population, but in close proximity to an international border with Niger.

### 2.5. Sample size

Overall, a total sample size of 600 children was required for this study. For each state, a sample size of 300 (150 children in each 6–9 and 36–47 month old age groups) was expected to provide sufficient precision to estimate seroprevalence to all three serotypes. This sample size calculation was based on the lowest expected seroprevalence value of 73% (for serotype 3 in 6–9 month old children, obtained from a similar survey conducted in Kano state in 2011 [6]), a 95% confidence level and a precision of ± 7.5%. A 10% inflation was applied to account for possible exclusions.

### 2.6. Eligibility criteria

Children fulfilling age requirements and residing within Borno or Yobe states for at least one month prior to the study, with a consenting parent or guardian, were eligible to participate, except those with: a) contraindication for venepuncture; b) serious acute or chronic illness which may require hospitalization; or c) diagnosed or suspected congenital immunodeficiency disorder in the subject or an immediate family member.

### 2.7. Enrolment and survey procedures

Ethical committee approvals were obtained from the Research Ethics Committee of University of Maiduguri Teaching Hospital and Ethical Review Committee of World Health Organization, Geneva and the study procedures were conducted following the principles of good clinical practices. The study enrolment took place between 11 January and 5 February 2016. Parents and guardians of children aged 6–9 or 36–47 months of age visiting the study clinic were approached for participation in this survey. If the child was determined to be eligible, informed consent was obtained from the parent or guardian. Once enrolled, the study physician administered a short questionnaire and physical examination to assess the child’s health and nutritional status. Detailed demographic and immunization history of the child was taken.

### 2.8. Blood collection and antibody testing procedures

Following the physical exam, a trained phlebotomist collected 1 ml of blood by venepuncture to test for serum neutralizing antibodies. Sera were sent to the Centers for Disease Control and Prevention (CDC) in Atlanta, where they were tested for levels of neutralizing antibody titres against poliovirus serotypes 1, 2 and 3. The tests were conducted in triplicate using a modified micro-neutralization assay [11, 12], with twofold serial dilutions of 1:8 to 1:1024. Antibody titers were reported on the log2 scale with the lower and upper limits of quantification at 2.5 and 10.5 log2, respectively. These were converted to reciprocal titers for analysis. Reciprocal antibody titres ≥8 were considered positive.

### 2.9. Routine and SIA doses

In Nigeria, RI doses of trivalent OPV (tOPV) were recommended at birth, 6, 10 and 14 weeks of age until April 2016, when the country switched to bOPV as part of the globally synchronized cessation of tOPV. Multiple SIAs/IPDs with either bOPV or tOPV targeting all children <5 years of age have been conducted in Borno and Yobe every year for >15 years. Children are administered OPV in SIAs irrespective of their immunization status. In this study, children 6–9 months of age could have been exposed to 1–2 bOPV doses and 3–4 tOPV doses through SIAs. Children 36–47 months of age could have been exposed to at least 20 bOPV and 11 tOPV doses through SIAs. In February 2015, one dose of IPV was introduced into RI in Borno and Yobe states. Therefore, all children 6–9 months had the potential to receive IPV in RI. Moreover, IPV was added to the OPV SIA in Borno and Yobe states in June-August 2014. All children aged 36–47 months would have been potentially exposed to this IPV SIA.

### 2.10. Definitions

Dose histories of OPV and IPV were based on parental recall as immunization cards were not available for most children enrolled in the survey. The doses received through RI and SIA were reported separately with all OPV and IPV doses considered except the final dose if it was administered on the day of blood collection. Measurements of height and weight were used to assess nutritional status and determine malnutrition (i.e. stunting and wasting) by comparing the child’s measurements to the WHO Child Growth Standards reference population based on the WHO Multicenter Growth Reference Study (MGRS) [13]. Nutritional status was classified into normal, moderate or severe based on the Z-score (i.e. the number of standard deviations a given data point lies from the mean). The classification was defined in terms of the following Z-scores: normal <2; moderate 2.0–2.99; and severe ≥3.

### 2.11. Data management and statistical analysis

The data collected in the questionnaires were double-entered into an initial database using CSPro software version 5.0 [14]. The association between dichotomous potential predictors and seroprevalence was assessed using Fisher’s exact tests. For ordinal variables, the Cochrane-Armitage test for trend was used to test for trend in seroprevalence across sub-groups. Logistic regression was used to estimate adjusted odds ratios and assess the association of risk factors with seroprevalence. All risk factors with P<0.1 in the univariable analysis were considered in the model. The most parsimonious yet best-fitting model based on the Akaike Information Criterion (AIC) was selected for each serotype. P-values <0.05 were considered statistically significant. Assessment of the additional impact of IPV on seropositivity in RI in 6-9-month-olds and IPV in SIAs in 36-47-month-olds for Borno and Yobe was conducted using Fisher’s exact tests. The anti-polio neutralizing antibody titer distributions were compared across age groups, states and IPV status (through RI and SIAs) using the Wilcoxon rank-sum test. For each age group, state and IPV status, reverse cumulative antibody titer distribution curves were prepared. The median anti-polio neutralizing antibody titers were estimated and 95% confidence intervals were calculated using bootstrapping with 10,000 replications. The analyses considering the impact of IPV were restricted to children with at least three prior OPV doses. All data analysis was conducted using R (R Foundation) version 3.2.3 (2015) [15].

## 3. Results

### 3.1. Final study sample

A total of 583 subjects were enrolled in the study, of whom 296 were from Borno and 287 were from Yobe. In Yobe, 139 and 148 children aged 6–9 and 36–47 months old, respectively, were enrolled. In Borno, 148 children were enrolled in each age group. Although the study participation was open to the entire state, most participants were from the local government areas (LGAs) of interest; 96% of participants for the Borno survey were from Jere and Maiduguri LGAs and 96% of participants for the Yobe study were from Nguru and Damaturu LGAs.

### 3.2. Characteristics of study subjects

The demographic information of the study participants is presented in Table 1. The proportion of male subjects was higher than female subjects, particularly in the 6–9 month age group in Borno state. There was a high prevalence of moderate and severe stunting among participants in both age groups and states, with 25–30% of children presenting with severe stunting. Yobe had higher RI dose history compared to Borno with 67% and 83% of subjects in Yobe reporting four OPV RI doses for children 6–9 and 36–47 months, respectively, compared to 45% and 56% of subjects in Borno for the two age groups. Moreover, coverage of one dose of IPV in RI in Yobe was double that in Borno (65% compared to 32%). In addition to having higher RI dose history, Yobe state also had higher SIA coverage, for both OPV dose history and IPV coverage.

**Table 1:** Demographic information of study children in Borno and Yobe States, North-Eastern Nigeria, 2016. SIA = supplementary immunization activity; RI = routine immunization; OPV = oral poliovirus vaccine; n = number of children; N = total number of children.

	Borno State		Yobe State	
Attribute	Age 6–9 months (n = 148)	Age 36–47 months (n = 148)	Age 6–9 months (n = 139)	Age 36–47 months (n = 148)
	n/N (%)	n/N (%)	n/N (%)	n/N (%)
**Gender**				
Female	58/148 (39.5)	67/148 (45.3)	65/139 (46.8)	74/148 (50.0)
Male	89/148 (60.5)	81/148 (54.7)	74/139 (53.2)	74/148 (50.0)
**Mother’s education**				
Primary or Less	111/141 (78.7)	109/146 (74.7)	98/138 (71.0)	95/147 (64.6)
Secondary/Tertiary	30/141 (21.3)	37/146 (25.3)	40/138 (29.0)	52/147 (35.4)
**Father’s education**				
Primary or Less	79/139 (56.8)	68/146 (46.6)	66/138 (47.8)	76/147 (51.7)
Secondary/Tertiary	60/139 (43.2)	78/146 (53.4)	72/138 (52.2)	71/147 (48.3)
**No. of children <5 years**				
1–2	110/140 (78.6)	112/142 (78.9)	83/136 (61.0)	102/148 (68.9)
>2	30/140 (21.4)	30/142 (21.1)	53/136 (39.0)	46/148 (31.1)
**Wasting**				
1-no	133/143 (93.0)	114/145 (78.6)	130/137 (94.9)	136/148 (91.9)
2-moderate	7/143 (4.9)	20/145 (13.8)	6/137 (4.4)	8/148 (5.4)
3-severe	3/143 (2.1)	11/145 (7.6)	1/137 (0.7)	4/148 (2.7)
**Stunting**				
1-no	79/143 (55.2)	76/146 (55.2)	80/138 (58.0)	88/148 (59.5)
2-moderate	22/143 (15.4)	30/146 (15.4)	22/138 (15.9)	22/148 (14.9)
3-severe	42/143 (29.4)	40/146 (29.4)	36/138 (26.1)	38/148 (25.7)
**Routine OPV doses**				
0	30/147 (20.4)	31/145 (21.4)	23/138 (16.7)	13/148 (8.8)
1	15/147 (10.2)	8/145 (5.5)	5/138 (3.6)	6/148 (4.1)
2	14/147 (9.5)	12/145 (8.3)	6/138 (4.4)	5/148 (3.4)
3	22/147 (15.0)	13/145 (9.0)	11/138 (8.0)	1/148 (0.7)
4	66/147 (44.9)	81/145 (55.9)	93/138 (67.4)	123/148 (83.1)
**SIAs OPV doses**				
0	17/148 (11.5)	7/147 (4.8)	20/137 (14.6)	2/145 (1.4)
1–3	52/148 (35.1)	13/147 (8.8)	36/137 (26.3)	6/145 (4.2)
4–6	61/148 (41.2)	27/147 (18.4)	56/137 (40.9)	12/145 (8.3)
≥7	18/148 (12.2)	100/147 (68.0)	25/137 (18.3)	125/145 (86.2)
**Total doses (RI and SIAs)**				
0	4/147 (2.7)	4/144 (2.8)	6/136 (4.4)	0/145 (0.0)
1–3	23/147 (15.7)	8/144 (5.6)	11/136 (8.1)	2/145 (1.4)
4–6	54/147 (36.7)	18/144 (12.5)	39/136 (28.7)	7/145 (4.8)
≥7	66/147 (44.9)	114/144 (79.2)	80/136 (58.8)	136/145 (93.8)
**IPV in RI**				
Yes	47/145 (32.4)	NA	86/133 (64.7)	NA
No	98/145 (67.6)	NA	47/133 (35.3)	NA
**IPV in SIA**				
Yes	1/146 (0.7)	55/143 (38.5)	8/138 (5.8)	69/135(51.1)
No	145/146 (99.3)	88/143 (61.5)	130/138 (94.2)	66/135 (48.9)

### 3.3 Anti-polio neutralizing antibody titers

In Borno, the anti-polio neutralizing antibody titers were consistently >900 for serotypes 1 and 2, with the lower values for serotype 3, particularly in the 36–47 month age group (median, 455 [95% confidence interval, CI: 277–1152]) (Table 2). There were no significant differences in antibody titer distributions between the 6–9 and 36–47 month age groups, for any of the serotypes (Table 2 and S1 Fig).

**Table 2:** Median reciprocal antibody titers by age group, poliovirus serotype and state.

		Type 1	Type 2	Type 3
State	Age Group	Median (95% CI)	Median (95% CI)	Median (95% CI)
Borno	6–9 mos	≥1448 (1152-≥1448)	1152 (724–1152)	724 (227–965)
	36–47 mos	1152 (1152-≥1448)	1152 (910-≥1448)	455 (277–1152)
Yobe^a^^c^	6–9 mos	1152 (576-≥1448)	910 (576-≥1448)	512 (288–1152)
	36–47 mos	1152 (910-≥1448)	910 (576–1152)	910 (576-≥1448)

^a^Significant difference in reciprocal antibody titer values between 6–9 and 36–47 mos age group for type 1 (α = 0.05) based on Wilcoxon rank-sum test

^b^Significant difference in reciprocal antibody titer values between 6–9 and 36–47 mos age group for type 2 (α = 0.05) based on Wilcoxon rank-sum test

^c^Significant difference in reciprocal antibody titer values between 6–9 and 36–47 mos age group for type 3 (α = 0.05) based on Wilcoxon rank-sum test.

In Yobe, the anti-polio neutralizing antibody titers were similar, albeit slightly lower than in Borno (except for serotype 3 in the 36–47 month age group) and there was a significant difference in antibody titers between the two age groups for serotypes 1 and 3 (Table 2 and S1 Fig).

### 3.4. Seroprevalence

**Fig 2:**
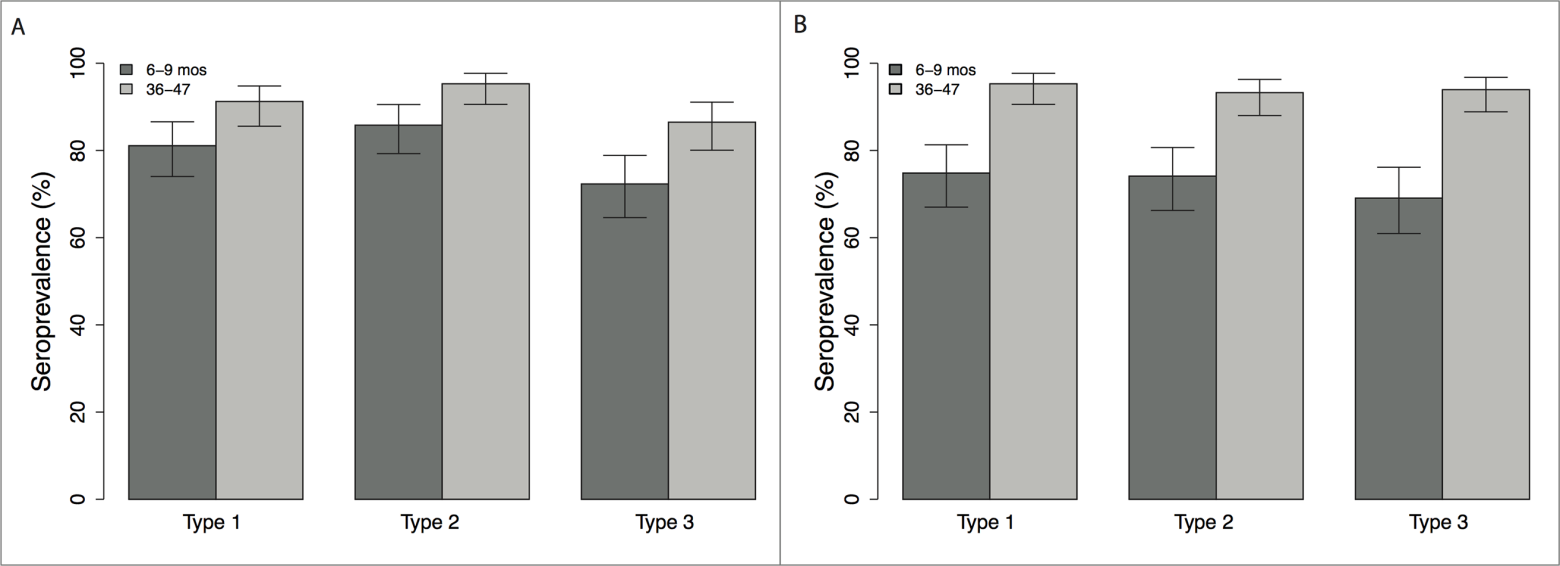
Seroprevalence and 95% confidence intervals, by poliovirus serotype and age group, Borno and Yobe States, North-Eastern Nigeria, 2016. (A) Borno; and (B) Yobe.

In Borno, the seroprevalence in 6-9-month-old children to serotypes 1, 2 and 3 was 81% (95% CI, 74–87%), 86% (79–91%) and 72% (65–79%), respectively (Fig 2A). The seroprevalence in the 36-47-month-old children was higher at 91% (86–95%), 95% (91–98%) and 87% (80–91%).

In Yobe, the seroprevalence in the younger age group (6–9 month olds) was lower than in Borno, with estimates of 75% (67–81%), 74% (66–81%) and 69% (61–76%) for serotypes 1, 2 and 3, respectively (Fig 2B). In the older age group (36–47 month olds), seroprevalence in Yobe was comparable to that in Borno for serotype 1 (95% (91–98%)) and serotype 2 (93% (88–96%)), with slightly higher levels for serotype-3 (94% (89–97%)).

### 3.5. Risk factors for seroprevalence

The univariable analysis of seroprevalence by demographic characteristics and immunization history for 6-9-month-old children is presented in Table 3 and Table 4 for Borno and Yobe states, respectively. The only consistently significant determinants of seroprevalence across both states and the three serotypes were RI and total OPV doses. In Yobe, father’s education was also a significant determinant of seroprevalence, but only for serotypes 2 and 3. A similar trend was observed for serotype 1; however, it was not statistically significant. In multivariable analysis, RI doses were the only consistently significant determinant of seropositivity for all three serotypes (Table 5). IPV in RI was found to be a significant determinant of seropositivity and anti-polio neutralizing antibody titers in 6-9-month-olds for serotypes 1 and 3 (Table 6, Table 7 and S2 Fig). A positive trend was observed for type 2 but the results were not statistically significant. In contrast, IPV in SIAs in 36-47-month-olds in Borno and Yobe did not demonstrate a significant impact on seropositivity, despite a similar albeit less pronounced positive trend (Table 6). However, a significant relationship was found for serotypes 1 and 3 when considering anti-polio neutralizing antibody titers (Table 7 and S2 Fig).

**Table 3:** Univariate analysis of predictors of seropositivity among subjects 6–9 months by demographic or other attributes, Borno State, North-Eastern Nigeria, 2016. SIA = supplementary immunization activity; RI = routine immunization; OPV = oral poliovirus vaccine; n = number of children; N = total number of children.

		Type 1		Type 2		Type 3	
	N	n	% (95% CI)	n	% (95% CI)	n	% (95% CI)
Seroprevalence	148	120	81.1 (74.0–86.6)	127	85.8 (79.3–90.5)	107	72.3 (64.6–78.9)
Predictors							
**Gender**^d^							
Female	58	46	79.3 (67.2–87.8)	48	82.8 (71.1–90.4)	42	72.4 (59.8–82.3)
Male	89	73	82.0 (72.8–88.6)	78	87.6 (79.2–93.0)	64	71.9 (61.8–80.2)
**Mother’s education**^d^							
Primary or Less	111	88	79.3 (70.8–85.8)	92	82.9 (74.8–88.8)	80	72.1 (63.1–79.6)
Secondary/Tertiary	30	25	83.3 (66.4–92.7)	28	93.3 (78.7–98.2)	22	73.3 (55.6–85.8)
**Father’s education**^d^							
Primary or Less	79	65	82.3 (72.4–89.1)	65	82.3 (72.4–89.1)	58	73.4 (62.8–81.9)
Secondary/Tertiary	60	46	76.7 (64.6–85.6)	54	90.0 (79.9–95.3)	43	71.7 (59.2–81.5)
**No. of children <5 years**^d^							
1–2	110	86	78.2 (69.6–84.9)	96	87.3 (79.8–92.3)	79	71.8 (62.8–79.4)
>2	30	26	86.7 (70.3–94.7)	24	80.0 (62.7–90.5)	23	76.7 (59.1–88.2)
**Wasting**^e^							
1-no	133	109	82.0 (74.6–87.6)	116	87.2 (80.5–91.9)	97	72.9 (64.8–79.8)
2-moderate	7	5	71.4 (35.9–91.8)	5	71.4 (35.9–91.8)	4	57.1 (25.1–84.2)
3-severe	3	2	66.7 (20.8–93.9)	2	66.7 (20.8–93.9)	2	66.7 (20.8–93.9)
**Stunting**^e^							
1-no	79	67	84.8 (75.3–91.1)	70	88.6 (79.8–93.9)	56	70.9 (60.1–79.8)
2-moderate	22	17	77.3 (56.6–89.9)	16	72.7 (51.9–86.9)	15	68.2 (47.3–83.6)
3-severe	42	32	76.2 (61.5–86.5)	37	88.1 (75.0–94.8)	32	76.2 (61.5–86.5)
**Routine OPV doses**^e^^a^^b^^c^							
0	30	19	63.3 (45.5–78.1)	17	56.7 (39.2–72.6)	17	56.7 (39.2–72.6)
1	15	12	80.0 (54.8–93.0)	13	86.7 (62.1–96.3)	6	40.0 (19.8–64.3)
2	14	11	78.6 (52.4–92.4)	14	100.0 (78.5–100.0)	11	78.6 (52.4–92.4)
3	22	18	81.8 (61.5–92.7)	21	95.5 (78.2–99.2)	18	81.8 (61.5–92.7)
4	66	59	89.4 (79.7–94.8)	61	92.4 (83.5–96.7)	54	81.8 (70.9–89.3)
**SIAs OPV doses**^e^							
0	17	11	64.7 (41.3–82.7)	13	76.5 (52.7–90.4)	10	58.8 (36.0–78.4)
1–3	52	42	80.8 (68.1–89.2)	42	80.8 (68.1–89.2)	34	65.4 (51.8–76.9)
4–6	61	50	82.0 (70.5–89.6)	55	90.2 (80.2–95.4)	47	77.1 (65.1–85.8)
≥7	18	17	94.4 (74.2–99.0)	17	94.4 (74.2–99.0)	16	88.9 (67.2–96.9)
**Total doses (RI and SIAs)**^e^^a^^b^^c^							
0	4	0	0.0 (0.0–49.0)	1	25.0 (4.6–69.9)	1	25.0 (4.6–69.9)
1–3	23	16	69.6 (49.1–84.4)	14	60.9 (40.8–77.8)	10	43.5 (25.6–63.2)
4–6	54	44	81.5 (69.2–89.6)	50	92.6 (82.5–97.1)	42	77.8 (65.1–86.8)
≥7	66	59	89.4 (79.7–94.8)	61	92.4 (83.5–96.7)	53	80.3 (69.2–88.1)

^a^Covariate significantly associated with seropositivity to type 1 for α = 0.05

^b^Covariate significantly associated with seropositivity to type 2 for α = 0.05

^c^Covariate significantly associated with seropositivity to type 3 for α = 0.05

^d^Fisher’s exact test

^e^Cochrane-Armitage test for trend.

**Table 4:** Univariate analysis of predictors of seropositivity among subjects 6–9 months by demographic or other attributes, Yobe State, North-Eastern Nigeria, 2016. SIA = supplementary immunization activity; RI = routine immunization; OPV = oral poliovirus vaccine; n = number of children; N = total number of children.

		Type 1		Type 2		Type 3	
	N	n	% (95% CI)	n	% (95% CI)	n	% (95% CI)
Seroprevalence	139	104	74.8 (67.0–81.3)	103	74.1 (66.2–80.7)	96	69.1 (61.0–76.2)
Predictors							
**Gender**^d^							
Female	65	44	67.7 (55.6–77.8)	45	69.2 (57.2–79.1)	43	66.2 (54.0–76.5)
Male	74	60	81.1 (70.7–88.4)	58	78.4 (67.7–86.2)	53	71.6 (60.5–80.6)
**Mother’s education**^d^							
Primary or Less	98	70	71.4 (61.8–79.4)	69	70.4 (60.7–78.5)	65	66.3 (56.5–74.9)
Secondary/Tertiary	40	33	82.5 (68.1–91.3)	34	85.0 (70.9–92.9)	31	77.5 (62.5–87.7)
**Father’s education**^d^^b^^c^							
Primary or Less	66	45	68.2 (56.2–78.2)	44	66.7 (54.7–76.8)	39	59.1 (47.1–70.1)
Secondary/Tertiary	72	58	80.6 (70.0–88.1)	59	81.9 (71.5–89.1)	57	79.2 (68.4–87.0)
**No. of children <5 years**^d^							
1–2	83	64	77.1 (67.0–84.8)	62	74.7 (64.4–82.8)	60	72.3 (61.8–80.8)
>2	53	37	69.8 (56.5–80.5)	39	73.6 (60.4–83.6)	35	66.0 (52.6–77.3)
**Wasting**^e^							
1-no	130	96	73.9 (65.7–80.6)	95	73.1 (64.9–80.0)	88	67.7 (59.3–75.1)
2-moderate	6	6	100.0 (61.0–100.0)	6	100.0 (61.0–100.0)	6	100.0 (61.0–100.0)
3-severe	1	1	100.0 (20.7–100.0)	1	100.0 (20.7–100.0)	1	100.0 (20.7–100.0)
**Stunting**^e^							
1-no	80	59	73.8 (63.2–82.1)	59	73.8 (63.2–82.1)	56	70.0 (59.2–78.9)
2-moderate	22	17	77.3 (56.6–89.9)	16	72.7 (51.9–86.9)	16	72.7 (51.9–86.9)
3-severe	36	28	77.8 (61.9–88.3)	28	77.8 (61.9–88.3)	24	66.7 (50.3–79.8)
**Routine OPV doses**^e^^a^^b^^c^							
0	23	10	43.5 (25.6–63.2)	9	39.1 (22.2–59.2)	9	39.1 (22.2–59.2)
1	5	4	80.0 (37.6–96.4)	4	80.0 (37.6–96.4)	4	80.0 (37.6–96.4)
2	6	3	50.0 (18.8–81.2)	4	66.7 (30.0–90.3)	3	50.0 (18.8–81.2)
3	11	6	54.6 (28.0–78.7)	7	63.6 (35.4–84.8)	4	36.4 (15.2–64.6)
4	93	81	87.1 (78.8–92.5)	79	85.0 (76.3–90.8)	76	81.7 (72.7–88.3)
**SIAs OPV doses**^e^							
0	20	12	60.0 (38.7–78.1)	13	65.0 (43.3–81.9)	11	55.0 (34.2–74.2)
1–3	36	25	69.4 (53.1–82.0)	27	75.0 (58.9–86.3)	23	63.9 (47.6–77.5)
4–6	56	47	83.9 (72.2–91.3)	43	76.8 (64.2–85.9)	43	76.8 (64.2–85.9)
≥7	25	19	76.0 (56.6–88.5)	20	80.0 (60.9–91.1)	18	72.0 (52.4–85.7)
**Total doses (RI and SIAs)**^e^^a^^b^^c^							
0	6	1	16.7 (2.0–56.4)	1	16.7 (2.0–56.4)	1	16.7 (2.0–56.4)
1–3	11	5	45.5 (21.3–72.0)	6	54.6 (28.0–78.7)	4	36.4 (15.2–64.6)
4–6	39	32	82.1 (67.3–91.0)	33	84.6 (70.3–92.8)	30	76.9 (61.7–87.4)
≥7	80	65	81.3 (71.3–88.3)	63	78.8 (68.6–86.3)	60	75.0 (64.5–83.2)

^a^Covariate significantly associated with seropositivity to type 1 for α = 0.05

^b^Covariate significantly associated with seropositivity to type 2 for α = 0.05

^c^Covariate significantly associated with seropositivity to type 3 for α = 0.05

^d^Fisher’s exact test

^e^Cochrane-Armitage test for trend.

**Table 5:** Multivariate analysis of predictors of seropositivity among subjects 6–9 months by demographic or other attributes Borno and Yobe States, North-Eastern Nigeria, 2013. RI = routine immunization; P-value indicates significance based on best fitting model for each poliovirus serotype.

			Type 1		Type 2		Type 3	
State	Covariate	Comparison	OR (95% CI)	P-value	OR (95% CI)	P-value	OR (95% CI)	P-value
Borno	RI Dose (continuous)	Each additional dose	1.44 (1.12–1.87)	0.005	1.84 (1.36–2.58)	<0.001	1.56 (1.24–1.98)	0.001
Yobe	RI Dose (continuous)	Each additional dose	1.66 (1.31–2.13)	<0.001	1.65 (1.30–2.11)	<0.001	1.16 (0.78–1.75)	<0.001

**Table 6:** Additional impact of IPV on seropositivity in Borno and Yobe States, North-Eastern Nigeria, 2013. IPV in routine immunisation (RI) in 6–9 month olds and IPV in supplementary immunisation activities (SIAs) in 36–47 month olds. Analysis restricted to children receiving at least 3 OPV doses. SIA = supplementary immunization activity; RI = routine immunization; OPV = oral poliovirus vaccine; IPV = inactivated poliovirus vaccine; n = number of children; N = total number of children.

		Type 1				Type 2			Type 3	
Method	IPV	n/N	% (95% CI)	p-value	n/N	% (95% CI)	p-value	n/N	% (95% CI)	p-value
RI	No	85/112	75.9 (67.2–82.9)	0.010	92/112	82.1 (74.0–88.1)	0.090	77/112	68.7 (59.7–76.6)	0.015
	Yes	118/133	88.7 (82.2–93.0)		120/133	90.2 (84.0–94.2)		110/133	82.7 (75.4–88.2)	
SIA	No	133/143	93.0 (87.6–96.1)	0.427	133/143	93.0 (87.6–96.1)	0.271	128/143	89.5 (83.4–93.5)	0.520
	Yes	114/119	95.8 (90.5–98.2)		115/119	96.6 (91.7–98.7)		110/119	92.4 (86.2–96.0)	

**Table 7:** Median reciprocal antibody titers by IPV status.

		Type 1	Type 2	Type 3
Method	IPV	Median (95% CI)	Median (95% CI)	Median (95% CI)
RI^a^^b^	No	910 (576-≥1448)	1152 (724-≥1448)	362 (144–724)
	Yes	≥1448 (1152-≥1448)	≥1448 (1152-≥1448)	1152 (724-≥1448)
SIA^a^^b^	No	1152 (910–1152)	910 (576–1152)	455 (256–724)
	Yes	≥1448 (1152-≥1448)	910 (724–1152)	1024 (576-≥1448)

^a^Significant difference in reciprocal antibody titer values between IPV and no IPV group for type 1 (α = 0.05) based on Wilcoxon rank-sum test

^b^Significant difference in reciprocal antibody titer values between IPV and no IPV group for type 3 (α = 0.05) based on Wilcoxon rank-sum test.

## 4. Discussion

We assessed the seroprevalence to poliovirus serotypes 1, 2 and 3, in 6-9- and 36-47-month-old children in accessible areas of Borno and Yobe States of Nigeria, in early 2016. The seroprevalence in the accessible health-care-seeking parts of both states was suboptimal and likely below the population threshold to interrupt transmission. These findings indicate remaining issues with immunization coverage in these States.

The seroprevalence in Borno was found to be higher in the younger age group when compared to Yobe but slightly lower for the older age group (with the exception of serotype 2 which was comparable in the two states). The higher seroprevalence in the 6-9-month-old children in Borno is unexpected given the lower dose history for both RI and SIAs with OPV and IPV when compared to Yobe. This could be due to the continued undetected circulation of WPV1 and cVDPV2 in Borno contributing to infection-induced immunity, as the neutralization assay does not differentiate between antibodies induced by natural infection or vaccination.

The anti-polio neutralizing antibody titers were consistently high across serotypes and states, indicating substantial impact of OPV and IPV in this population. The sustained level of antibodies in the 36–47 month age group is indicative of repeat SIAs, as in the absence of SIAs titers are expected to decline with age following doses administered through RI services. The lowest antibody levels were for serotype 3, particularly in Borno. This further supports the presence of persistent circulation of WPV1 resulting in infection-induced immunity, as the efficacy of tOPV and bOPV for serotypes 1 and 3 are comparable (albeit slightly lower for serotype 3)[16–18].

In both Borno and Yobe, RI OPV doses were significantly associated with seropositivity to all three serotypes. In addition to the positive impact of RI OPV doses on seropositivity, one dose of IPV in RI demonstrated a significant impact on both seroprevalence and anti-polio neutralizing antibodies of serotypes 1 and 3; a similar trend was observed for serotype 2; however, it was not statistically significant. The association between RI OPV doses and seropositivity has been consistently demonstrated across poliovirus serotypes in seroprevalence surveys in Northern Nigeria [5, 6]. This evidence supports the impact of a strong RI system in protecting children from poliomyelitis and the added benefit of an IPV dose through RI services on immune protection from paralysis.

In contrast to the role of RI doses (either OPV or IPV) on seroprevalence, doses administered in SIAs did not have a significant impact on seroprevalence for any of the three poliovirus serotypes. Despite the lack of significant finding, there was a consistent positive trend with increased number of SIA OPV doses, total OPV doses and the presence of an IPV SIA dose. The impact of SIAs on seroprevalence is more pronounced at older age groups; however, in the older age group seroprevalence was already relatively high and therefore it is more difficult to demonstrate a significant impact. A significant impact of IPV in SIAs was demonstrated when considering levels of anti-polio neutralizing antibodies for serotypes 1 and 3. The lack of significant finding for serotype 2 could be explained, in part, by the variable exposure to tOPV and bOPV doses in the population through SIAs which would differentially boost serotypes 1 and 3 more than 2.

In the multivariable analysis, there were no demographic or nutritional risk factors demonstrating a significant association with seroprevalence in either Borno or Yobe. The lack of association between demographic factors, particularly maternal or paternal education, contrasts with previous seroprevalence surveys in Northern Nigeria, Egypt and India [5, 6, 8, 19]. The relationship for nutritional status is consistent with the seroprevalence surveys in Northern Nigeria, despite evidence that malnutrition and concurrent enteric infections may inhibit OPV replication and immune response in immunised children [20].

There are study limitations that must be mentioned. Firstly, as this was a facility-based study, it only represents the health-seeking and accessible populations and not those in the inaccessible areas. However, given the context of the security and access issues in these states, this was the only safe and practical option available. Secondly, the number of OPV and IPV doses reported was based on parent recall which may be subject to error, particularly at high dose numbers (as accurate recall at higher dose numbers becomes more difficult). Therefore, recall bias is likely a greater concern for SIA doses than RI doses, which may have contributed to the lack of significant findings for OPV and IPV SIAs. Moreover, we were unable to explore the impact of IPV through SIAs on the younger age group due to the low coverage of IPV in this age group. The comparison between IPV and non-IPV groups was restricted to children with ≥3 OPV doses to ensure comparable samples. Repeating the analysis for children with <3 OPV doses was not possible due to very low numbers with IPV in this group. Finally, as this was a seroprevalence study and therefore only measured individual protection to poliomyelitis, further studies assessing mucosal immunity in the population (or similar populations) are important to assess the level of mucosal protection given its importance on poliovirus transmission.

This study found that vaccination with OPV and IPV had a substantial impact on seroprevalence in Borno and Yobe. Given the importance of vaccination with OPV and/or IPV on seroprevalence, the program must find innovative ways to secure access and effectively vaccinate the unreached children. In response to the recent WPV1 and cVDPV2 detections, an outbreak response spanning large areas in Northern Nigeria and the neighbouring Lake Chad region has been launched in the form of multiple bOPV and monovalent serotype 2 OPV campaigns. To assess the impact of multiple campaigns on population immunity, we intend to repeat the seroprevalence survey in Borno in 2017. In addition to the health-facility-based sample, we plan to include internally displaced person (IDP) camp populations to assess immunity of the populations recently moved from inaccessible to accessible areas.

## Supporting information

S1 Fig Reverse cumulative poliovirus reciprocal antibody curves by age group, serotype and state. Colour of lines correspond to age group, with red and grey corresponding to 6–9 and 36–47 mos, respectively. (A-C) Serotype 1, 2 and 3 in Borno State. (D-F) Serotype 1, 2 and 3 in Yobe State.
(JPG)

S2 Fig Reverse cumulative poliovirus reciprocal antibody curves by inactivated poliovirus vaccine (IPV) status and serotype. Colour of lines correspond to IPV status, with red and grey corresponding to IPV and No IPV, respectively. (A-C) Serotype 1, 2 and 3 comparing IPV status in routine immunization (RI). (B) Serotype 1, 2 and 3 comparing IPV status in supplementary immunization activities (SIA).
(JPG)

S1 Dataset Dataset of included children in the seroprevalence survey for Borno and Yobe States, North-Eastern Nigeria. (XLSX)

S1 File Data dictionary for dataset of included children in the seroprevalence survey for Borno and Yobe States, North-Eastern Nigeria. (DOCX)

S1 Questionnaire Seroprevalence survey questionnaire for Borno and Yobe States, North-Eastern Nigeria. (PDF)
