# Supplementary figures and images for: Survey of poliovirus antibodies in Borno and Yobe States, North-Eastern Nigeria
Source: PLoS One. 2017 Sep 26;12(9):e0185284. doi: 10.1371/journal.pone.0185284 (PMC5614605; doi:10.1371/journal.pone.0185284)

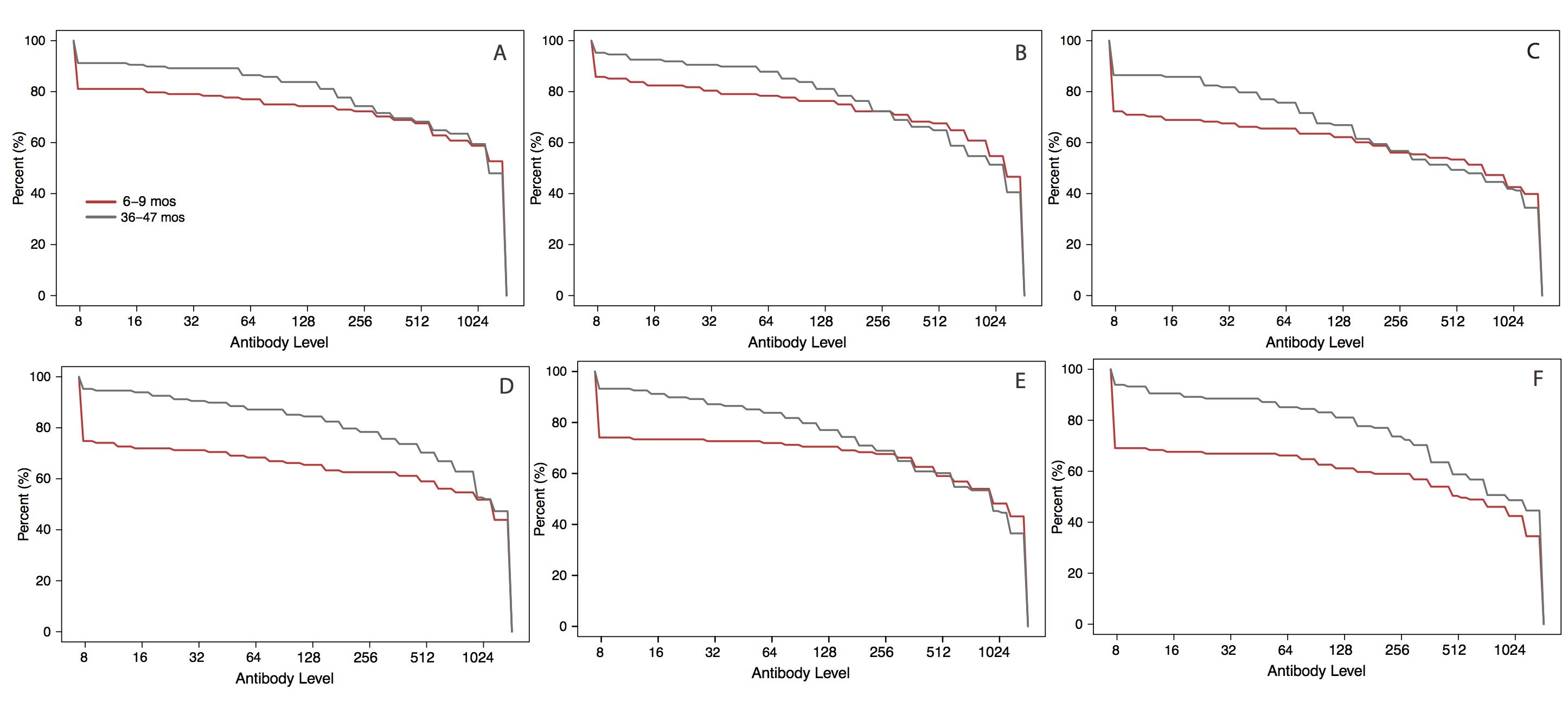

Supplement: S1 Fig — Colour of lines correspond to age group, with red and grey corresponding to 6–9 and 36–47 mos, respectively. (A-C) Serotype 1, 2 and 3 in Borno State. (D-F) Serotype 1, 2 and 3 in Yobe State. (JPG) [file pone.0185284.s001.jpg]

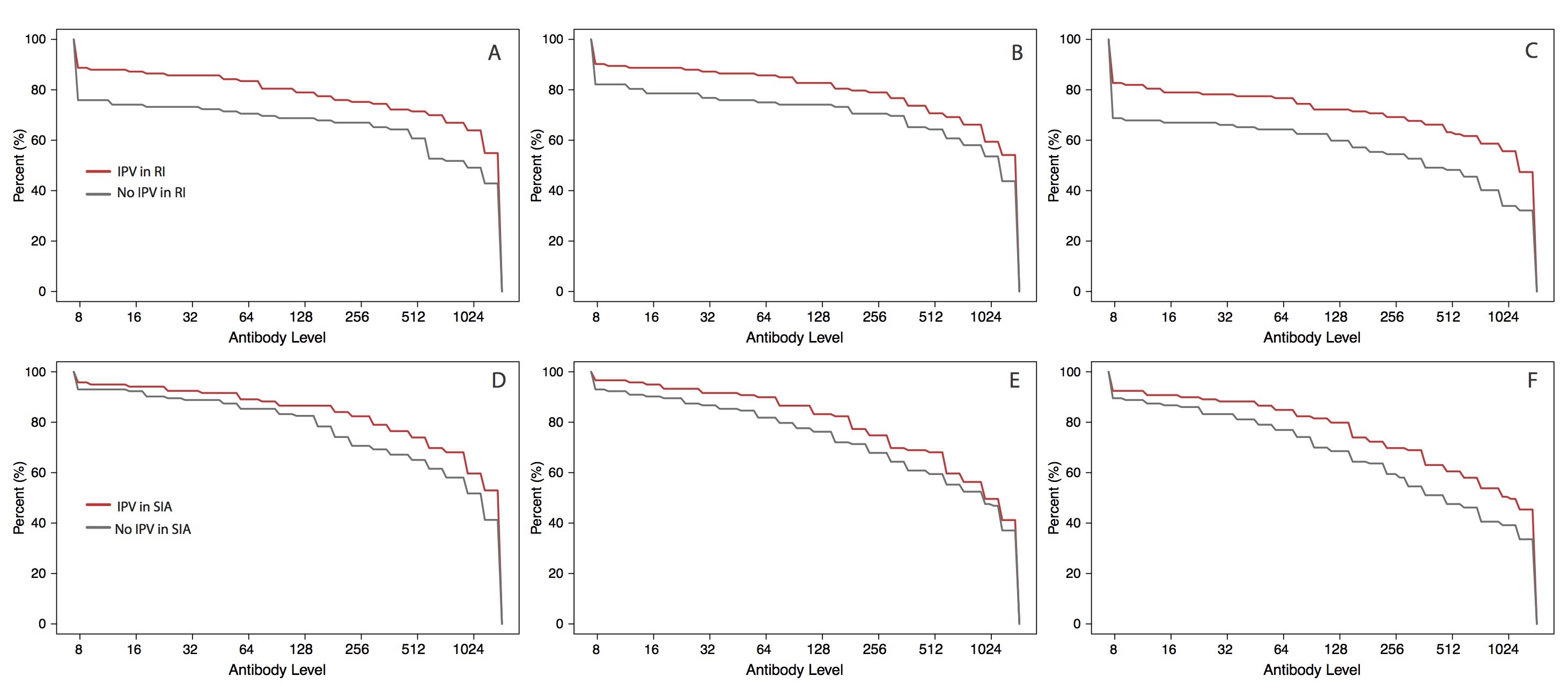

Supplement: S2 Fig — Colour of lines correspond to IPV status, with red and grey corresponding to IPV and No IPV, respectively. (A-C) Serotype 1, 2 and 3 comparing IPV status in routine immunization (RI). (B) Serotype 1, 2 and 3 comparing IPV status in supplementary immunization activities (SIA). (JPG) [file pone.0185284.s002.jpg]
