# Supplementary material for: Survey of poliovirus antibodies in Borno and Yobe States, North-Eastern Nigeria
Source: PLoS One. 2017 Sep 26;12(9):e0185284. doi: 10.1371/journal.pone.0185284 (PMC5614605; doi:10.1371/journal.pone.0185284)
Supplement: S1 File — (DOCX) [file pone.0185284.s004.docx]

**Borno and Yobe SPS data dictionary**

| **Variable** | **Definition** |
| --- | --- |
| ID | Child identifying number |
| SEX | Sex of child  1 = Female  2 = Male |
| STATE | 1 = Borno  2 = Yobe |
| AGE_GROUP | 1 = 6-9 months  2 = 36-47 months |
| WEIGHT | Weight of child in kilograms (kg) |
| HEIGHT | Height of child in centimeters (cm) |
| N_CHILDREN | Number of children <5 years in household  1 = 1-2 children  2 = >2 children |
| MOTHER_S_EDUCATION | Mother’s education  0 = Primary or Less  1 = Secondary/Tertiary |
| FATHER_S_EDUCATION | Father’s education  0 = Primary or Less  1 = Secondary/Tertiary |
| IPV_IN_CAMPAIGN | IPV received by child through supplementary immunization activities (SIAs)  1 = Yes  0 = No |
| IPV_IN_RI | IPV received by child through routine immunization (RI)  1 = Yes  0 = No |
| Sabin.1 | Anti-polio neutralizing antibody titer against serotype-1 |
| Sabin.2 | Anti-polio neutralizing antibody titer against serotype-2 |
| Sabin.3 | Anti-polio neutralizing antibody titer against serotype-3 |
| RI_doses | Number of OPV doses child received through RI |
| SIA_doses | Number of OPV doses child received through SIAs |
