# Supplementary material for: Survey of poliovirus antibodies in Borno and Yobe States, North-Eastern Nigeria
Source: PLoS One. 2017 Sep 26;12(9):e0185284. doi: 10.1371/journal.pone.0185284 (PMC5614605; doi:10.1371/journal.pone.0185284)
Supplement: S1 Questionnaire — (PDF) [file pone.0185284.s005.pdf]

Polio seroprevalence survey in Borno and Yobe, Nigeria, 2015

Questionnaire - Part I
(Eligibility assessment)

Fill in or encircle the option applicable

Study Site & Code ________________________Date ____/____/________ (DD/MM/YYYY)

Identification data

1. Name of the child____ 2. Sex F / M

3. DOB If known ____ / _____ / _____ 4. Age ___ Years____ Months ____Days

5. Mother's name____

6. Father's name____

7. Father's occupation ____

8. Mother's occupation ____

9. Address with landmarks ____

____

10. LGA_____________________ Ward _________________Ward Code ___________________

Settlement Name__________________________ Settlement ID _____________________________

Alternative Settlement Name ____

10 (a). Does this settlement figure in the list of settlements of the area provided and has been matched: Y/N

10 (b). If not, why ____

11. Name and Phone number of the care giver: ____

12. Eligibility assessment

- Is the child within the required age range (birth record / best estimate) Yes / No
  (encircle the age range) (6-9 M / 36-47 M)
- Does the residential address of child fall in Borno/Yobe? Yes / No
- Has the child stayed at this address continuously for last one month? Yes / No
- Have the parent/guardian provided consent for child's participation? Yes / No
- Any known coagulation disorders? Yes / No

- Any evidence of congenital immunodeficiency disorder? Yes / No

Physical examination

Weight in Kg.(up to 2 decimal points) ______:______ Height/Length (cm) ___________

Temp (ºC) ______

Physical Examination ____

Based upon the physical exam, does the child has any serious illness or requires hospitalization?

Yes / No, If yes, specify ___________________________and then blood sample is not to be taken)

Finally meets all Eligibility Criteria Yes / No (Blood sample to be taken only if Yes)

If eligible, allot an ID Number. **ID NUMBER _______**

(ID Number will be alphanumeric including the study team series A / B / C / D and the serial number for that team in three digits, e.g. 1st subject enrolled by Team A will have ID as A 001)

Questionnaire –Part II
(Data collection from enrolled subjects only)

Fill in or encircle the option applicable

ID NUMBER ____________ **Parent/Guardian's initials______**
(first three alphabets)

Age group (Please encircle) I. (6-9 months) / II. (36-47 months)

Socioeconomic History

1. Number of children <5 years in household (including study subject if <5 years): ___

2. Mother's education (encircle one or more):

Never attended school / Quranic Islamic school / Primary / Secondary/Tertiary/ PG

3. Father's education (encircle one or more):

Never attended school / Quranic Islamic school / Primary / Secondary/Tertiary/ PG

Immunization History:

4. DPT/Pentavalent (encircle whatever is applicable) Unknown / Nil (if the case be)

(Write Y/N for each dose): 1st dose ______ 2nd dose _______ 3rd dose __________

5. Number of routine immunization (RI) OPV doses: Unknown / Nil (if the case be)

Birth dose_________1st dose __________ 2nd dose__________ 3rd dose________
(Write Y/N for each dose)

Total No of OPV doses in RI ______________ (count all Y and write the number)

6. Place where RI doses were administered (Encircle): Government facility / Outreach sessions / Private sector / other

7. Source of information for RI doses: Immunization Card / Recall /other?

8. If RI doses are not commensurate with age, reason/s for missing vaccination (encircle one or more):

a. No services in the area b. Erratic services c. Parental ignorance

d. Parental neglect e. Parental concern on safety f. Parental disbelief in immunization

g. others (specify)……………………………..

9. Number of OPV doses received through IPD (SIA campaign): Encircle one option

Unknown / 0 /1 / 2 / 3 / 4 / 5 / 6 / 7-10 / >10

10. Source of information: Any record / Recall

11. If IPD/SIA doses are not commensurate with age and number of SIAs in the area, reason/s for missing doses

a. No services in the area b. Erratic services c. Parental ignorance

d. Parental neglect e. Parental concern on safety f. Parental disbelief in immunization

g. others (specify)………………………………..

12. OPV dose in last SIA given: Y / N / NA, if no, reason (encircle) – a / b / c / d / e / f / g

13. Date of last OPV (encircle RI or SIA): ___ / ____ / ____

14. Total OPV doses (RI + SIA) RI doses from Q.5______SIA doses from Q.9________ Add to write the Total (in Number if up to 6, if >6, write the range like 7-10 or >10)__________

15. Did this child receive a dose of IPV during the July/Augst2014 campaign

1. Yes 2. No 3. Not sure

15 (a). Source of information: Any record / Recall

16. Did this child receive a dose of IPV under RI?

1 Yes 2 No 3 Not sure

16 (a). Source of information: Any record / Recall

Blood Collection

17. Blood collected: Yes / No, If not collected, specify reason: ___________________________

18. Time of collection: ___________ (hh:mm)

19. Quantity sufficient ($\geq$ 1 ml): Yes / No.

20. Correct ID label fixed on the Vacutainer: Yes / No

21. Any untoward incidents during observation: Yes / No, if yes describe_______________________

Health benefits/advice

22. Provision of a mosquito net? Yes / No
23. Provision of soap packet? Yes / No
24. Health education on malaria prevention and hygiene Yes / No
25. Referral to immunization clinic if required? Yes / No

Name of the research physician filling the questionnaire –

Signature of the research physician filling the questionnaire –

Date - (DD-MM-YYYY)
